# Longitudinal changes in cognitive ability following traumatic brain injury: a systematic review and meta-analysis

**DOI:** 10.1093/braincomms/fcag149

**Published:** 2026-05-04

**Authors:** Maria Loizidou, Christina Christoforou, Fofi Constantinidou

**Affiliations:** Center for Applied Neuroscience, University of Cyprus, Nicosia, 1678, Cyprus; Department of Psychology, University of Cyprus, Nicosia, 1678, Cyprus; Center for Applied Neuroscience, University of Cyprus, Nicosia, 1678, Cyprus; Department of Psychology, University of Cyprus, Nicosia, 1678, Cyprus; Center for Applied Neuroscience, University of Cyprus, Nicosia, 1678, Cyprus; Department of Psychology, University of Cyprus, Nicosia, 1678, Cyprus

**Keywords:** cognitive change, traumatic brain injury, longitudinal, executive function

## Abstract

The longitudinal impact of traumatic brain injury on cognitive functioning has been widely studied, though findings remain inconsistent. This may be due to variations in injury severity between participants, and the timing of assessments between studies. This meta-analysis aims to address these limitations by providing an overview of longitudinal cognitive changes in moderate-severe traumatic brain injury cohorts. This meta-analysis was conducted in accordance with the Preferred Reporting Items for Systematic Reviews and Meta-Analysis Protocols and was registered with PROSPERO (CRD42024562211). PubMed, EMBASE via Ovid and APAPsychInfo via EBSCOHost were last searched in August 2025 and were screened based on pre-established eligibility criteria, regardless of publication date, including longitudinal cognitive assessment in moderate-severe traumatic brain injury cohorts. Studies without follow-up assessments in at least 3 months after the baseline assessment were excluded. Separate random effects meta-analyses were conducted for each assessed cognitive domain (memory, processing speed, verbal ability, visuospatial ability, executive functioning), due to the expected variability in the change trajectories between them. Where appropriate, meta-regression analyses using a random effects maximum likelihood estimator examined the impact of assessment timing on cognitive change. Risk of bias was assessed using the Newcastle Ottawa Scale and Critical Appraisal Skills Programme. Of 9481 original articles, 21 met the eligibility criteria and included a total of 972 participants. Significant improvements were found in memory, processing speed, verbal and visuospatial ability, with effect sizes ranging from 0.27 to 0.38. However, longitudinal assessment of executive functioning did not indicate significant changes (*P* = 0.712). Meta-regression revealed that shorter durations between injury and baseline assessment predicted greater improvements in processing speed (*β* = −0.01, SE = 0.00, 95%CI [−0.009, −0.005], *z* = −6.18, *P* < 0.001) and memory (*β* = −0.005, SE = 0.00, 95%CI [−0.007, −0.003], *z* = −6.31, *P* < 0.001). Findings suggest consistent, subtle improvements across most cognitive domains, with potential non-linear trajectories, especially for memory and processing speed. However, it is acknowledged that statistically significant improvements have limited clinical significance. Insignificant changes in executive functioning are discussed in relation to potential differing profiles of dysfunction following traumatic brain injury.

## Teaser

Traumatic brain injury leads to long-term cognitive challenges. However, variability in injury characteristics and assessment timings leads to uncertainty regarding the progression of these difficulties. This review indicates that survivors generally improve in memory, processing speed, verbal and visuospatial abilities, while executive function remains unchanged from acute to chronic stages.

## Graphical Abstract

**Graphical Abstract:**
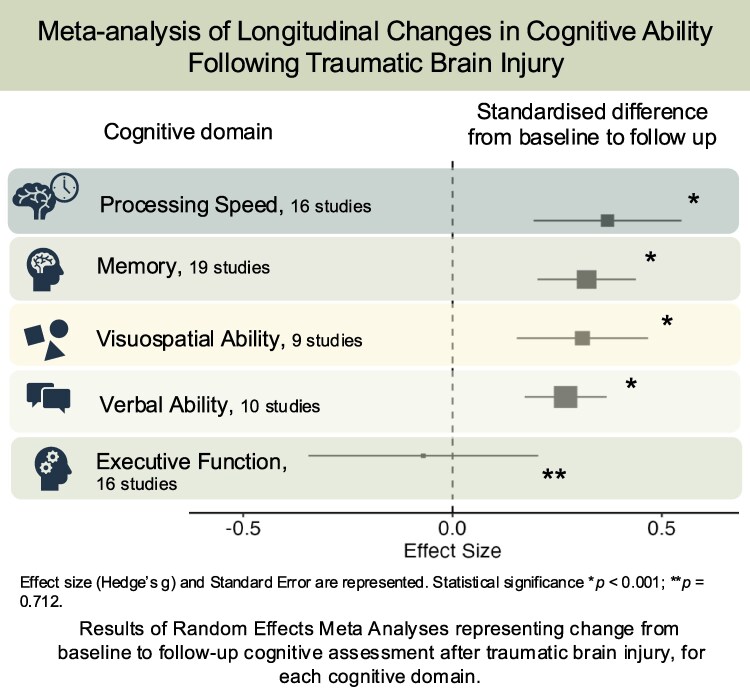

## Introduction

The substantial decline of cognitive abilities following traumatic brain injury (TBI) is associated with reduced functional ability, as well as social and occupational quality of life, making TBI a challenging and multifaceted condition to cope with.^1,2^ TBI is the result of head trauma due to an external force, with the most common injury mechanisms in adults being vehicle accidents or falls.^3^ The trauma itself can increase intracranial pressure and lead to haemorrhage and axonal injury, which impair the transportation of organelles, resulting in intracellular damage.^4,5^ Following the acute injury, a cascade of secondary pathophysiological changes starts to occur, which are more profound in individuals with more severe injuries and are associated with poorer long-term outcomes.^6-8^

An array of factors can moderate one’s susceptibility to cognitive decline following TBI, such as genetic predisposition to neurodegeneration,^9,10^ premorbid ability, social support,^11,12^ quality of care and access to rehabilitation.^1,13^ Exploration of cognitive changes following moderate-severe TBI shows a non-linear trajectory, with the most rapid improvements occurring within 6 months of the injury, and slowly reaching a plateau at impaired levels.^14^ This explains the remarkable gains observed in acute injury cohorts, relative to insignificant longitudinal improvements in the cognitive performance of chronic survivors.^15,16^ Investigation of change trajectories is critical, because early cognitive outcomes may act as more reliable predictors of chronic functional outcome, than injury variables and demographics.^17-19^

Adding to the aforementioned complexities, each cognitive domain appears to have a distinct trajectory, which may be partially related to its differential susceptibility towards age-related decline.^20,21^ In a cohort of 48 moderate-severe TBI survivors, 41% presented with a decline in their memory performance and 19% showed a decline in executive functioning (EF) tasks, from 12 to 30 months post-injury. On the contrary, 14% showed an improvement in memory and 22% in EF tasks, within the same time window.^9^ Therefore, when considering cognitive changes following TBI, it might be redundant to think of cognition as a uniform function.

The most recent meta-analysis on longitudinal changes in cognitive ability after TBI compared the number of studies reporting improvement or decline across cognitive domains, within predefined time windows.^22^ While this approach offers a broad overview, it does not provide a statistically robust estimate of the overall direction of findings. Furthermore, it included cohorts with mild TBI and sports-related concussions,^22^ creating a highly heterogeneous sample that differs markedly from moderate-to-severe TBI in cognitive profile.^23^ The current meta-analysis addresses these limitations by: (i) focusing on a homogenous sample of moderate-severe TBI cohorts, and (ii) using random-effects models and meta-regression to assess the overall change in cognitive ability from baseline to follow-up assessments and examine how injury chronicity may shape these outcomes.

## Materials and methods

The current meta-analysis was conducted in accordance with the Preferred Reporting Items for Systematic Reviews and Meta-Analyses (PRISMA) criteria.^24^ A review protocol was registered with PROSPERO on 25 July 2024 (CRD42024562211). Minor changes were made to the registered protocol during the screening phase to improve methodological consistency and in no way affect the primary objectives. These amendments are detailed in the Eligibility Criteria section.

### Eligibility criteria

The following inclusion criteria were considered: (i) study type included longitudinal, cohort, case–control, cross-sectional studies or randomized control trials (untreated arms only); (ii) at least 60% of the cohort assessed as having moderate-severe TBI, as defined by objective measures (e.g. Glasgow Coma Scale < 13, post-traumatic amnesia > 24 h)^25^; (iii) age at injury over 16 years old; (iv) no comorbid psychiatric conditions; (v) re-assessment of the same cohort at least once, no sooner than 3 months after the baseline assessment; (vi) published in a peer-reviewed journal, in the English language. Inclusion of criterion (iii) was based on international guidelines where adult treatment protocols are followed for people over 16 years of age.^26^

Exclusion criteria included: (i) paediatric cohorts, cadaver studies, animal studies; (ii) stroke and/or acquired brain injury cohorts; (iii) exclusively subjective measures for TBI severity and/or cognitive ability (e.g. self-report); (iv) exclusive use of screening tools to assess cognition (e.g. Montreal Cognitive Assessment—MoCA); (v) reviews, case studies, case series. Preprints were not eligible as they are typically not peer reviewed.

Compared to the original PROSPERO protocol, inclusion and exclusion criteria were made more stringent to enhance methodological rigour. Specifically, inclusion criterion (iv) and exclusion criterion (iv) were added to minimize potential confounding variables that could influence the outcomes of the meta-analysis. Regarding inclusion criterion (ii), the threshold of 60% moderate-severe TBI participants was selected for reasons of pragmatism, to balance sample homogeneity with sufficient statistical power for meta-analyses. This criterion was applied only in cases where authors compiled results across injury severities. More details on sample compositions are provided in Supplementary Table 1.

### Search strategy

PubMed, Embase via Ovid and APA PsychInfo via EBSCOHost databases were searched for eligible articles on 26 July 2024. The search was repeated on 4 August 2025 to capture any additional articles published within that time frame. There was no restriction on the article publication date. A comprehensive search strategy was constructed following a preliminary search that involved careful inspection of keywords in the relevant literature. The detailed search strategy is provided in Supplementary material. Reference lists of included articles were manually searched to identify additional studies.

### Data management and selection process

Literature search results were imported into the Rayyan AI software^27^ for deduplication. The remaining articles were assessed for eligibility based on title and/or abstract by authors M.L. and C.C. Potentially relevant articles or those with ambiguous titles and unavailable abstracts were retrieved for full text. Articles for full-text retrieval were blindly assessed by M.L. and C.C. Any disagreement regarding the eligibility of articles was resolved with consensus among the two primary authors.

### Data extraction and quality assessment

Eligible articles were marked on Rayyan and data were extracted independently by the two reviewers on an Excel spreadsheet (Version 16.93) with the following order: author(s), year, title, DOI, study design, study aim, country, sample size, demographic information (gender, age/age at injury, education), TBI severity and assessment, loss of consciousness and/or post traumatic amnesia, other TBI injury variables, timing of cognitive assessments (relative to injury), cognitive tests used and the corresponding cognitive domains, cognitive outcomes (at each time point), functional/psychological outcomes if applicable and key findings.

Risk of bias was assessed blindly by two independent reviewers (ML, CC). Quantitative non-randomized studies were assessed using the Newcastle-Ottawa Scale (NOS) for cohort or case–control studies, as appropriate (Suppl. Tables 2 and 3).^28^ Despite the lack of universally established cut-off scores for the NOS, scores equal to or higher than six are considered to be high quality.^29^ For randomized studies, risk of bias was assessed using the Critical Appraisals Skills Programme (CASP) for Randomized Control Trials (Suppl. Table 4).^30^ All included studies were assessed to be of adequate quality.

### Cognitive domains

The following cognitive domains were assessed across studies: memory, processing speed, verbal ability, visuospatial ability and EF. Not all included studies assessed all the above cognitive domains. There was variability in the classification of cognitive domains assessed by each test. To overcome this, a comprehensive list of the cognitive tests used, and the corresponding domains assessed was devised based on thorough examination of the literature, and prior to statistical analyses (Suppl. Tables 5–9). It is, however, acknowledged that performance on cognitive tests relies on more than one cognitive domain.^25^

### Calculation of effect sizes

Means and standard deviations from each study, at each time point, were extracted, as discussed in the Data Extraction and Quality Assessment section. If this information was not available directly, an effect size was calculated based on other statistical information available, such as mean change scores, *t*-test, or *P*-values. This process was facilitated using Comprehensive Meta-Analysis (CMA) (version 4), as it allows for effect size calculation from a range of different statistical tests.^31^ In studies where only standardized values were reported, and this process could not be facilitated, authors were contacted to recover raw data. Due to the small sample sizes in TBI literature, Hedge’s *g* was the chosen effect size and was calculated based on the mean change of cognitive scores from initial assessment (baseline) to the latest follow-up, for each cognitive domain.^32,33^ If multiple cognitive tests were employed to assess the same cognitive domain, then effect sizes were pooled to create a composite score. Pooled effect sizes were calculated using the inverse variance weighted average method.^34^ The specific method was selected due to its ability to minimize uncertainty and wide use in cognitive research.^35,36^

For analyses in the domains of memory, processing speed, language ability and visuospatial ability, positive Hedges’ *g* values indicated improvement in cognitive performance from baseline to follow-up, while negative values indicated decline. For measures where higher scores represented worse performance (e.g. completion time or error count), scores were inverted before effect size computation to ensure consistent directionality across studies. For the domain of EF, where higher scores represented worse performance, negative Hedge’s *g* values represent improvement in cognitive ability from baseline to follow-up.

### Statistical analyses

Separate random-effects meta-analyses were conducted to examine longitudinal cognitive changes, for each cognitive domain (memory, processing speed, verbal ability, visuospatial ability, EF), in moderate-severe TBI. A random effects model using Restricted Maximum Likelihood (REML) was chosen as it is deemed more realistic and appropriate for longitudinal samples.^37^ Statistical analyses were facilitated using JASP version 0.19.3.^38^

Evaluation of meta-analyses included tests of heterogeneity, sensitivity analyses and publication bias. Heterogeneity was assessed by Cochran Q statistic (statistically significant heterogeneity set at *P* < 0.10), *I*^2^ statistic (< 25% mild heterogeneity, 25–50% moderate heterogeneity, > 50% large heterogeneity) and *τ*^2^ representing the variance of the true effect sizes.^37,39^ A leave-one-out procedure was conducted as part of sensitivity analyses, to investigate the impact of each individual study.^37^ Publication bias was assessed via visual inspection of funnel plots and the Egger’s test, which was considered statistically significant at *P* < 0.10.^40^

### Meta-regression analyses

Meta-regression models were further employed where appropriate to explain significant between-study heterogeneity. To avoid overfitting, only one predictor was entered in the model and was selected in advance based on the current literature.^37^ Time since injury at the baseline assessment was selected as the predictor, due to the large variability in the timing of assessments in the included studies, and evidence of non-linear change trajectories following TBI.^14,18^

## Results

**Figure 1:**
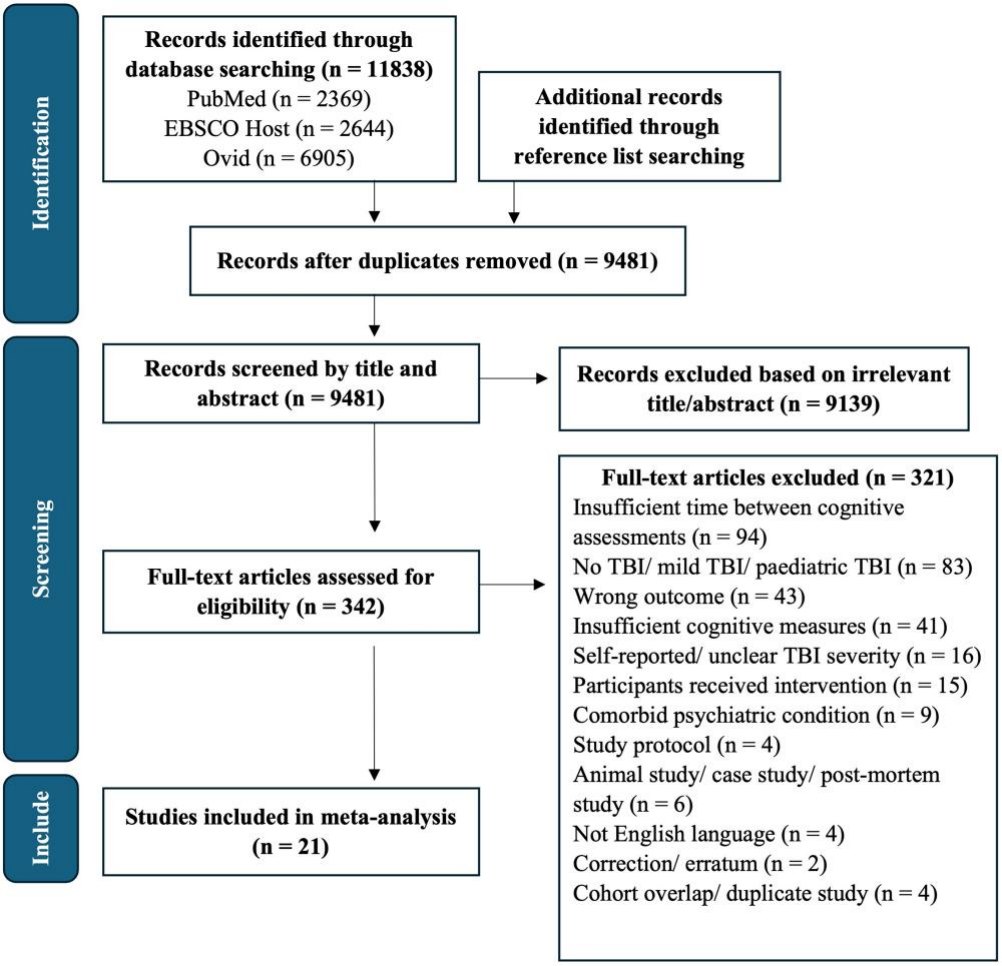
PRISMA flow diagram.

All articles published until 4 August 2025 were considered for this meta-analysis. The search yielded 11 838 articles across the three databases. After deduplication, 9481 unique articles remained for title and/or abstract screening. A total of 342 articles were retrieved in full-text, and of those, 21 were eligible for inclusion. Two articles were found via the reference list search of included articles. Two eligible studies were excluded as they assessed a sub-cohort of participants reported in another included study.^41^ This process is summarized in Fig. 1.

For the memory domain, 13 effect size estimates were obtained by pooling.^16,18,19,21,42-50^ For processing speed, six estimates were obtained by pooling.^16,19,42,46,51,52^ Seven estimates were obtained by pooling for verbal ability,^16,18,42,43,46,47,49^ four estimates for EF^15,16,18,48^ and four for visuospatial ability.^16,42,46,49^

### Characteristics of included studies

Twelve studies were conducted in America; eight studies were conducted in the USA, two in Brazil and two in Canada. Four studies were conducted in Australia and one in New Zealand. Three studies were conducted in Europe (Norway, Sweden/Iceland, Romania). One study was conducted in Asia (Malaysia). A summary of individual studies is provided in Supplementary Table 1.

Inclusion of male participants ranged from 60% to 88% across cohorts. Mean age at injury spanned from 28 years old to 52 years old and was relatively homogenous across cohorts (*M*_age_ = 35.89 years old, SD = 7.24). These demographics are consistent with epidemiological systematic review evidence and are considered representative of TBI cohorts.^3^ Sample sizes for the included cohorts ranged from 11 to 106 participants (*M* = 45.13, SD = 28.87), with a total of 972 participants. Baseline assessments ranged from 0 months to 10 years after the injury (*M* = 15.6 months, SD = 33.03). Latest follow-up ranged from the subacute (6 months post-injury) to the chronic phase (24 years post-injury) (*M* = 45.52 months, SD = 66.03).

### Meta-analyses

Results of the meta-analyses are reported for each cognitive domain separately, in Table 1. Forest plots are presented in each corresponding section. Overall, the longitudinal cognitive changes observed following moderate-severe TBI, in memory, processing speed, verbal and visuospatial ability, except for EF, were statistically significant. Sensitivity and heterogeneity analyses are reported in Table 2 for each of the cognitive domains and discussed further in the corresponding sections. A formal GRADE assessment to assess confidence in the expected results was not performed due to the predominantly observational nature of included studies, which limits the applicability of standard GRADE criteria for certainty of evidence.^53^

**Table 1:** Results of random-effects model meta-analyses exploring longitudinal cognitive changes following moderate-severe TBI

Cognitive domain	Number of studies	*g* (SE)	95%CI	*z*	*P*
Memory	19	0.32 (0.06)	[0.199, 0.440]	5.20	<0.001
Processing speed	16	0.38 (0.09)	[0.202, 0.555]	4.20	<0.001
Verbal ability^a^	10	0.27 (0.05)	[0.178, 0.354]	5.90	<0.001
Verbal ability	11	0.33 (0.07)	[0.199, 0.461]	4.94	<0.001
Visuospatial ability	9	0.31 (0.08)	[0.151, 0.465]	3.85	<0.001
Executive function	16	−0.06 (0.15)	[−0.356, 0.243]	−0.37	0.712

^a^Outliers excluded from analyses.

**Table 2:** Heterogeneity measures for the random-effects meta-analyses exploring longitudinal cognitive changes following moderate-severe TBI

Cognitive domain	Number of studies	*τ* ^2^	*I* ^2^ (%)	*Q*	*Q*_df_	*Q_p_*
Memory	19	0.06	86.56	122.06	18	<0.001
Processing speed	16	0.11	85.76	98.96	15	<0.001
Verbal ability^a^	10	0.01	34.68	12.35	9	0.194
Verbal ability	11	0.03	69.36	28.75	10	<0.001
Visuospatial ability	9	0.04	69.38	23.13	8	0.003
Executive function	16	0.34	93.96	159.05	15	<0.001

^a^Outliers excluded from analyses; *τ*^2^ = estimate of the variance of true effect sizes; *I*^2^ = amount of variance that is due to true effects and not sampling error; *Q* = tests the hypothesis that all included studies share a common effect size, significance set at *α* = 0.100.

### Memory

**Figure 2:**
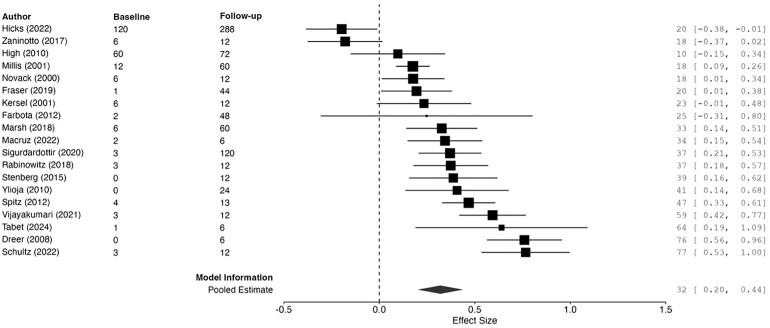
**Forest plot summarizing pooled estimates of change from baseline to follow-up memory assessment (diamond shape), using REML**. The model was significant, *g* = 0.32, 95%CI [0.20, 0.44], *z* = 5.20, *P* < 0.001. Each data point represents one study (author name and year shown) with size of black squares corresponding to study weight in the meta-analysis. ‘Baseline’ and ‘follow-up’ correspond to months since injury at baseline and latest memory assessment. Larger effect sizes correspond to greater improvements in memory ability. Effect sizes and 95% CIs are represented.

Nineteen studies included measures of memory.^16,18,19,21,42-50,52,54-58^ The overall model was significant (Table 1), indicating a small positive mean change from initial to latest memory assessment (Fig. 2). There was no publication bias as assessed via the Egger’s test, which was non-significant (*t* = 0.85, *P* = 0.408). The funnel plot under the REML was symmetrical, with data points clustered at the top, suggesting small standard errors (Suppl. Fig. 1).

A meta-regression model with REML estimation was fitted, with time since injury at baseline as the predictor, to explain the large between-study heterogeneity (Table 2). Although the model somewhat improved between-study heterogeneity, a significant amount remained, *Q*(17) = 82.23, *P* < 0.001. The intercept was significant, *β* = 0.36, SE = 0.02, *z* = 15.03, *P* < 0.001. Time since injury at initial assessment was a significant, negative predictor of memory ability, *β* = −0.005, SE = 0.00, 95%CI [−0.007, −0.003], *z* = −6.31, *P* < 0.001. Therefore, earlier assessments were associated with significantly larger improvements in memory at follow-up. However, the significant remaining heterogeneity suggests the critical influence of additional predictors not considered in the model.

Sixteen studies conducted memory assessments within the first 6 months after the injury.^16,18,19,43-45,47-50,52,54-58^ Neuroimaging studies showed greater whole brain volume loss correlating with poorer working memory performance, 2 months post-injury.^54^ However, there were significant improvements on memory measures between the baseline and the follow-up assessment.^16,43,47,55,56,58^ Similarly, repeat assessments at 3, 6 and 12 months post-injury indicated a linear trajectory of improvement.^48^ Still, evidence consistently showed that despite any improvements, performance of TBI survivors remained impaired relative to healthy controls.^19,43,49,55,57^

Sixteen of the included studies conducted assessments after the first year of injury.^18,19,21,42,43,45-50,52,54-56,58^ Comparison of baseline and follow-up outcomes obtained exclusively after the first year of injury showed a pattern of stability rather than improvement. At 10 years post-injury, a small subset of individuals experienced a decline in their memory ability, whilst the majority remained stable rather than continuing to improve.^18^ However, chronic improvements might be subject to test sensitivity, with a higher percentage of participants noting improvements on simpler memory tasks (e.g. Logical Memory), rather than more demanding tasks such as the digit span.^42,46^

### Processing speed

**Figure 3:**
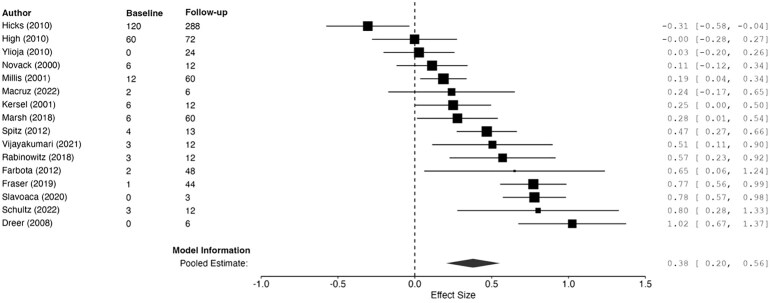
**Forest plot summarizing pooled estimates of change from baseline to follow-up processing speed assessment (diamond shape) using REML**. The model was significant, *g* = 0.38, 95%CI [0.20, 0.56], *z* = 4.20, *P* < 0.001. Each data point represents one study (author name and year shown) with size of black squares corresponding to study weight in the meta-analysis. ‘Baseline’ and ‘follow-up’ correspond to months since injury at baseline and latest processing speed assessment. Larger effect sizes correspond to greater improvements in processing speed ability. Effect sizes and 95% CIs are represented.

Sixteen studies included measures of processing speed.^16,19,21,42-49,51,52,54,55,58^ There was significant heterogeneity between studies (Table 2). The funnel plot under REML was relatively symmetrical (Suppl. Fig. 2), and there was no publication bias as indicated by the Egger’s test (*t* = 0.68, *P* = 0.51). The overall model was significant (Table 1), indicating a small mean improvement in processing speed ability from baseline to the latest follow-up assessment (Fig. 3).

Due to the large between-study heterogeneity, a meta-regression model with REML estimation was fitted, with time since injury at baseline as the predictor. Although the model improved between-study heterogeneity, a significant amount of heterogeneity remained, *Q*(14) = 60.78, *P* < 0.001. The intercept was significant, *β* = 0.44, SE = 0.04, *z* = 12.20, *P* < 0.001. Time since injury at baseline assessment was a significant, negative predictor of processing speed, *β* = −0.01, SE = 0.00, 95%CI [−0.009, −0.005], *z* = −6.18, *P* < 0.001. Therefore, a shorter duration between injury and baseline assessment was associated with larger improvements in processing speed between the assessed time points. Still, a significant amount of heterogeneity remained unexplained, suggesting the critical influence of additional predictors not considered in the model.

Thirteen studies assessed processing speed within the first 6 months after the injury.^16,19,43-45,47-49,51,52,54,55,58^ Shortly after the injury, processing speed of TBI survivors was significantly slower than that of controls, yet with significant within-group improvements observed within the first 6 months post-injury.^16,19,51^ Still, TBI survivors continued to function at an impaired level relative to their healthy counterparts.^16,19,54,55^ Slower processing speed was also associated with greater white matter volume atrophy.^44,54^

Thirteen studies conducted assessments after the first year of injury.^19,21,42,43,45-49,52,54,55,58^ Improvements in ability were evident by 12 months post-injury, when TBI survivors had significantly faster processing speed, compared to their performance at the baseline assessment.^45,47,49^ However, such improvements were subject to significant individual variability^49^ and in some cases were not retained beyond 12 months post-injury.^42^ Exploration of other influential variables revealed that improvements reached a plateau and remained stable among younger survivors, whereas older survivors were more likely to experience a decline.^48^

Closer inspection of impairment rates at various time points indicated that whereas 80% of one cohort was not impaired at the initial assessment (12 months post-injury), only 72% were unimpaired at follow-up (5 years post-injury). Thus, ∼8% of the sample declined within that 4-year window.^46^ In other cohorts, significant improvements in processing speed were seen even 5 years post-injury.^45^ Comparison with healthy controls indicated no significant differences in the rate of decline, suggesting that the observed decline in ability likely reflects a healthy ageing process evident in both TBI and healthy control participants.^21^

### Executive function

**Figure 4:**
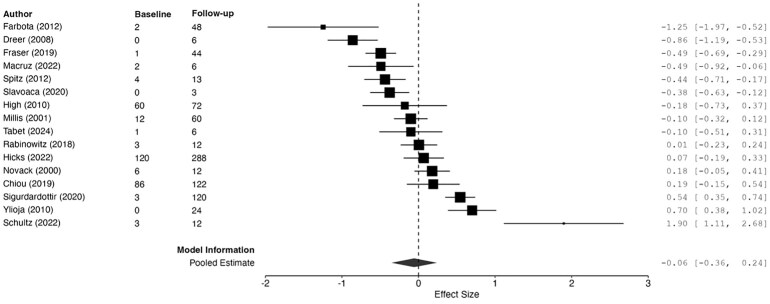
**Forest plot summarizing pooled estimates of change from baseline to follow-up assessment of executive functioning (diamond shape), using REML**. The model was not significant, *g* = −0.06, 95%CI [0.36, 0.24], *z* = −0.37, *P* 0.712. Each data point represents one study (author name and year shown) with size of black squares corresponding to study weight in the meta-analysis. ‘Baseline’ and ‘follow-up’ correspond to months since injury at baseline and latest EF assessment. Smaller effect sizes correspond to greater improvements in EF ability. Effect sizes and 95% CIs are represented.

Sixteen studies included measures of EF.^15,16,18,19,21,42,44,46-49,51,52,54,55,57^ The funnel plot under the REML was symmetrical (Suppl. Fig. 3), and the Egger’s test was not significant, *t* = −0.15, *P* = 0.887, suggesting no publication bias. A forest plot of included studies is presented in Fig. 4. The overall model was non-significant (Table 1), suggesting no significant change in longitudinal EF ability from baseline to follow-up assessment.

Thirteen studies assessed EF ability within the first 6 months of injury.^16,18,19,44,47-49,51,52,54,55,57^ Improvements on EF ability were observed between assessments conducted within the first year post-injury, across seven studies.^16,18,19,44,51,57,59^ Scores on the Trail Making Test B were significantly influenced by age at injury^44^ and were associated with total brain volume loss from 3 to 12 months post-injury.^59^ Notably, regardless of the degree of impairment in other cognitive functions, greater functional disability (Mayo-Portland Adaptability Inventory) was related to poorer EF scores in one cohort.^19^

Twelve studies included assessment of EF after the first year of injury, providing a comprehensive trajectory of EF ability following TBI.^15,18,19,21,42,46-49,52,54,55^ Longitudinal change of EF ability showed large individual variability, with Millis and colleagues^46^ estimating that 14.3% of their sample deteriorated from 1 to 5 years post-injury, whereas 22.1% had improved. Despite some significant improvements, TBI survivors remained impaired and performed either significantly worse than healthy controls, or below the normative cut-off scores, at 12 months post-injury,^19,47^ with relative stability over a 10-year period.^18,21^ Increases in fractional anisotropy in the superior longitudinal fasciculus and regions in the corpus callosum correlated with improvements on an EF task, between 7 years and 10 years post-injury, suggesting improvement in white matter structures that was reflected in EF performance.^15^ However, Hicks and colleagues argue that declining EF performance over a 12-year period followed a trajectory similar to that of healthy ageing in controls and was not related to the injury itself.^21^

### Verbal ability

**Figure 5:**
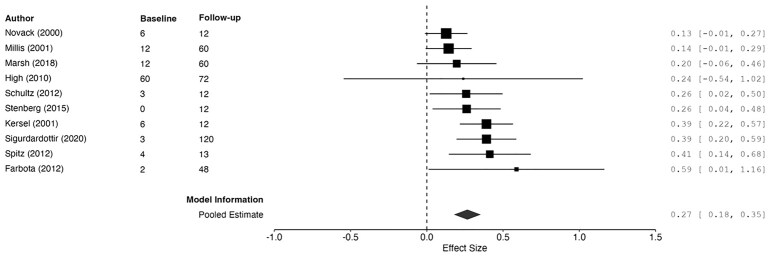
**Forest plot summarizing pooled estimates of change from baseline to follow-up assessment of verbal ability (diamond shape) after removal of the outlier study, using REML**. The model was significant, *g* = 0.27, 95%CI [0.18, 0.35], *z* = 5.90, *P* < 0.001. Each data point represents one study (author name and year shown) with size of black squares corresponding to study weight in the meta-analysis. ‘Baseline’ and ‘follow-up’ correspond to months since injury at baseline and latest verbal ability assessment. Larger effect sizes correspond to greater improvements in ability. Effect sizes and 95% CIs are represented.

Eleven studies measured longitudinal changes in verbal ability.^18,19,42,43,45-49,54,56^ The leave-one-out procedure indicated one possible outlier; meta-analysis results are reported both with and without the outlier study (Tables 1 and 2).^48^ After removal of the outlier, there was no longer significant between-study heterogeneity (Table 2). A forest plot of included studies after removal of the outlier study is presented in Fig. 5. On the funnel plot, there was clustering of data points at the top (Suppl. Fig. 4), and there was no publication bias based on the Egger’s test, *t* = 1.43, *P* = 0.191. Overall, there was a significant small positive change in verbal ability from baseline to follow-up assessment following TBI.

Eight studies had assessments within the first 6 months post-injury.^18,19,43,47,49,54,56^ Although TBI survivors overall showed improvements in verbal ability over the first year post-injury, they still presented with impairment relative to healthy controls.^19,43,47,54^ However, Novack and colleagues^47^ also note that the cognitive impairments observed in verbal ability were disproportionate to those seen in other domains, suggesting that tests relying on over-learned material (e.g. Vocabulary, Similarities—WAIS)^60^ may be insensitive to detect meaningful deficits.

Notably, different verbal abilities may be differently affected. Verbal comprehension assessed with the Token Test^61^ remained unimpaired over the first year post-injury.^49^ In a sample of 65 TBI survivors, 69% were impaired in the Controlled Oral Word Association Test^62^ of verbal fluency at 6 months, with 55% of the sample still being impaired by 12 months post-injury.^43^

All of the studies assessing verbal ability had assessments beyond the first year of injury.^18,19,42,43,45-47,49,54,56^ Any significant improvements observed between the first 6 months of injury and 4–5 years post-injury were argued to have small clinical significance.^45,46,54^ Acute performance was also associated with neuroimaging findings, where lower scores on the Controlled Oral Word Association Test^62^ at 1-year post-injury were associated with greater whole brain volume loss at 4 years post-injury.^54^ Comparisons between individuals who declined and those who improved in verbal measures revealed that only age at injury was a significant predictor of change.^46^

### Visuospatial ability

**Figure 6:**
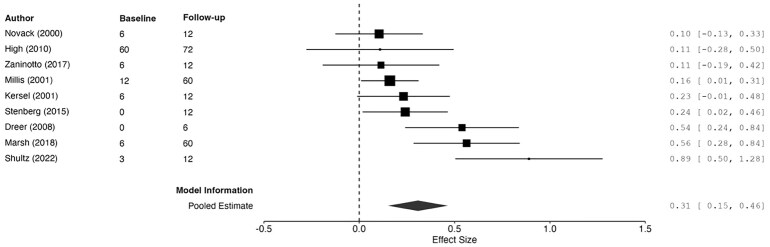
**Forest plot summarizing pooled estimates of change from baseline to follow-up visuospatial ability assessment (diamond shape), using REML**. The model was significant, *g* = 0.31, 95%CI [0.15, 0.46], *z* = 3.85, *P* < 0.001. Each data point represents one study (author name and year shown) with size of black squares corresponding to study weight in the meta-analysis. ‘Baseline’ and ‘follow-up’ correspond to months since injury at baseline and latest visuospatial ability assessment. Larger effect sizes correspond to greater improvements in ability. Effect sizes and 95% CIs are represented.

Nine studies included measures of visuospatial ability.^16,42,43,45-47,49,50,56^ A forest plot of included studies is presented in Fig. 6. There was some funnel plot asymmetry, with data points distributed on the left side (Suppl. Fig. 5). However, the Egger’s test was non-significant (*t* = 1.66, *P* = 0.140), suggesting no publication bias. Considering that between-study heterogeneity was also significant (Table 2), these differences likely reflect true differences between included studies. Overall, there was a small positive mean change in visuospatial ability from baseline to follow-up assessment. The small number of included studies did not permit further analyses to attempt to explain between-study heterogeneity.

Seven studies assessed visuospatial ability within the first 6 months of injury.^16,43,45,47,49,50,56^ When using the Block Design test,^60^ impairments at 6 months post-injury ranged from 18% to 40%, with 6–31% of survivors still being impaired by 12 months post-injury.^43,45^ Interestingly, use of the Block Design^60^ test showed borderline impairment from 6 to 12 months post-injury among those with more severe TBI, which was comparable to tasks requiring rapid responding, such as processing speed tasks.^47^ Evidence from another cohort showed significant improvement in visuospatial ability within the first 6 months post-injury.^16^

Eight studies conducted assessments of visuospatial ability beyond the first year of injury.^42,43,45-47,49,50,56^ Overall, changes were inconsistent across cohorts. Monthly assessments within the first 12 months post-injury revealed fluctuations, with 35% of the sample remaining severely impaired by 12 months post-injury.^49^ Another cohort found no significant differences in visuospatial ability between 6 and 12 months post-injury.^50^ In a cohort of 51 TBI survivors, 94% presented with no impairment by 5 years post-injury, with only 6% of the sample showing borderline impairment in their visuospatial ability.^45^ Further, Millis and colleagues^46^ found that Block Design performance showed the greatest improvement out of all the assessed cognitive domains (memory, processing speed, EF, verbal ability) from 1 to 5 years post-injury.^46^

## Discussion

The impact of moderate-severe TBI on cognitive function has been extensively studied, using various methodological approaches, often yielding mixed findings. Building on previous meta-analyses exploring the longitudinal cognitive changes in TBI, the present meta-analysis fills a critical gap that has not been previously addressed. Using random effects models and meta-regression analyses, we compared TBI cohorts with comparable injury severities to precisely explore the direction of cognitive changes following TBI and the exact influence of injury chronicity on assessment outcomes. As expected, overall results indicated significant improvements from baseline to follow-up assessment, albeit with small effect sizes across memory, processing speed, verbal and visuospatial ability. EF was the only cognitive domain that did not show a significant change between baseline and follow-up assessments.

For the memory domain, only two studies observed longitudinal decline,^21,50^ one of which also noted a decline in processing speed.^21^ Overall, studies with baseline assessments closer to the time of injury and chronic follow-ups were more likely to report greater improvement. This pattern was further supported by the meta-regression models, where a shorter time since injury predicted greater improvements in ability. These findings align with the broader literature emphasizing significant cognitive gains in the early phases after the injury, with stabilization typically occurring around 6 months post-injury, followed by possible decline in some cases.^9,63^

A similar pattern emerged for verbal and visuospatial ability, although these domains were assessed less frequently across the included studies, providing less confidence in the obtained findings. Notably, none of the included studies noted a decline in verbal and visuospatial abilities. Even when assessments occurred exclusively in the chronic phase, substantial improvements were still observed.^42,45,46^

With regard to verbal ability, it is critical to acknowledge the substantial influence of crystallized intelligence. Early factorial analyses by Woodcock indicated that tests of verbal ability load onto both a crystallized intelligence factor and a reading—writing factor.^64,65^ This could suggest that verbal ability is less impacted by the trauma and thus less susceptible to decline, which aligns with findings observing unimpaired or marginally impaired verbal comprehension and confrontation naming in the first year post-injury, relative to controls.^49^

Unlike the rest of the assessed cognitive domains, EF did not show significant longitudinal changes. It has been argued that EF performance is intertwined with other cognitive abilities, and indeed, evidence suggests moderate correlations between EF and memory tasks.^66,67^ Similarly, Zimmerman and colleagues^68^ identified two distinct clusters of EF dysfunction among TBI survivors—one characterized by deficits in ‘cognitive flexibility’, inhibition and speed of focused attention, and the second with additional deficits in working memory. Assessment of such EF clusters reveals differing change trajectories in the acute injury phase.^57^ Such findings are consistent with our theoretical understanding of EF, suggesting that distinct neural networks support these complementary EF processes.^66^ Thus, despite the relatively diffuse damage observed in moderate-severe TBI, the variability in executive dysfunction and thus improvement assessed via neuropsychological measures may stem from functional or structural differences in the underlying networks, which require a series of neuropsychological measures to be adequately captured.

However, it is important to acknowledge that performance in cognitive tasks is often criticized as having little ecological validity.^69^ Therefore, the longitudinal improvements observed in the current meta-analysis may have little functional importance for TBI survivors. Whereas this may be true for certain cognitive tasks, recent evidence suggests that processing speed outcomes (e.g. Trail Making Test) can be one of the strongest predictors of functional outcome.^70,71^ This may be due to the nature of processing speed ability, which can be more easily captured with simpler timed tasks that are quite representative of real-life demands. On the contrary, functions such as memory are thought to be much more complex, relying on past experiences, learning and intent, with current tools argued to be lacking in these sophisticated aspects and therefore in their real-life complexity.^69^ Despite standard neuropsychological tests having limited ecological validity in assessing a given cognitive domain, they can provide an accurate indication of overall functional ability, particularly for those who are experiencing greater disability. In fact, individuals who perform well on cognitive measures tend to have higher functional ability scores (e.g. Glasgow Outcome Scale Extended > 6), whereas those with poorer performance also present with higher levels of functional disability.^70^

### Directions for future research

Despite the wealth of research exploring trajectories of cognitive change following moderate-severe TBI, research has focused on memory, processing speed and EF assessments. Future research should aim to conduct more comprehensive neuropsychological assessments that include visuospatial and verbal abilities to better capture the cognitive profiles of TBI survivors. Additionally, the potential presence of several clusters of EF deficits following TBI could explain the lack of significant findings in this meta-analysis. Future research could employ neuroimaging techniques to explore potential executive dysfunction profiles and more comprehensive neuropsychological measures to capture the extent of EF.

## Conclusion

The current meta-analysis provides further evidence of partial cognitive improvement following moderate-severe TBI, with similar but modest improvements across memory, processing speed, verbal and visuospatial ability. Despite these improvements, it is important to acknowledge the substantial individual variability and range of factors that contribute to long-term outcomes such as premorbid ability, age and chronicity of the injury. Therefore, while group-level analyses indicated tendencies towards subtle improvements for certain cognitive domains, evidence from individual studies highlights the potential for chronic decline or stagnation. EF did not exhibit significant longitudinal change, likely due to its heterogeneity, individual variability and the limitations of standard neuropsychological assessments in effectively capturing this complex function. Taken together, our findings support conceptualization of TBI as a chronic and evolving condition, rather than a discrete episode. Therefore, we must remain vigilant to the importance of frequent cognitive monitoring and flexible rehabilitation planning that responds to individual needs.

### Limitations

It is vital to acknowledge that performance in each of the assessed cognitive domains is not entirely independent of one another, but rather interconnected. Whereas allocation of tests in cognitive domains was based on existing literature, several tests are thought to assess more than one cognitive domain. Therefore, interpretation of findings should be made with these considerations in mind. Further, despite quantitative results indicating longitudinal improvements in cognitive functioning from acute to chronic injury phases, it is important to emphasize that the clinical significance of such improvements is likely small, particularly for individuals in chronic stages of injury. Similarly, despite the use of meta-regression analyses to control for the variability in assessment timings between studies, the influence of this predictor, although significant, was small, suggesting the presence of other significant predictors in cognitive outcomes. Lastly, generalizability of our findings may be limited by the fact that only peer-reviewed studies in the English language were considered for this review. Despite the inclusion of studies from across the world, studies in America were predominantly included, and the exclusion of gray literature may further limit the generalizability of our conclusions.

## Supplementary Material

fcag149_Supplementary_Data

## Data Availability

The data supporting this meta-analysis were extracted from previously published studies. The compiled dataset is available from the corresponding author upon reasonable request.
