# Supplementary material for: Longitudinal changes in cognitive ability following traumatic brain injury: a systematic review and meta-analysis
Source: Brain Commun. 2026 May 4;8(3):fcag149. doi: 10.1093/braincomms/fcag149 (PMC13178829; doi:10.1093/braincomms/fcag149)
Supplement: fcag149_Supplementary_Data [file fcag149_supplementary_data.docx]

**Supplementary Material**

1. Search strategy for PubMed:

A.

1. Traumatic brain injury [MeSH]
2. Traumatic brain injury [Title/Abstract]
3. TBI [Text Word]
4. “brain injury” [Text Word]
5. “brain trauma” [Text Word]

Search: (((traumatic brain injury[MeSH terms]) OR ("traumatic brain injury"[Title/Abstract]) OR TBI[text word] OR ("brain injury"[text word]) OR ("brain trauma"[text word]))

B.

1. “cognitive abilit*” [Title/Abstract]
2. Cognition [MeSH]
3. “cognitive assess*” [Title/Abstract]
4. “cognitive performance” [Text Word]
5. “executive function*” [Title/Abstract]
6. “neurocognitive performance” [Text Word]
7. “cognitive domain*” [Title/Abstract]

Search: (("cognitive abilit*"[title/abstract]) OR (cognition[MeSH terms]) OR ("cognitive assess*"[title/abstract]) OR ("cognitive performance" [text word]) OR ("executive function*"[title/abstract]) OR ("neurocognitive performance"[text word]) OR ("cognitive domain*" [title/abstract])))

C.

1. “cognitive change*” [Title/Abstract]
2. “cognitive decline*” [Title/Abstract]
3. “cognitive difficult*” [Text Word]
4. “cognitive outcome*” [Title/Abstract]
5. “neurocognitive dysfunction” [Text Word]
6. longitudinal [Title/Abstract]
7. progress* [Title/Abstract]
8. chronic [Title/Abstract]
9. “cognitive impairment” [Title/Abstract]
10. neurodegen* [Text Word]

Search: ("cognitive change*"[Title/Abstract] OR "cognitive decline*"[Title/Abstract] OR "cognitive difficult*"[Text Word] OR "cognitive outcome*"[Title/Abstract] OR "neurocognitive dysfunction"[Text Word] OR "longitudinal"[Title/Abstract] OR progress*[title/abstract] OR chronic [title/abstract] OR "cognitive impairment"[title/abstract] OR neurodegen*[text word])

Query box: (((traumatic brain injury[MeSH terms]) OR ("traumatic brain injury"[Title/Abstract]) OR TBI[text word] OR ("brain injury"[text word]) OR ("brain trauma"[text word])) AND (("cognitive abilit*"[title/abstract]) OR (cognition[MeSH terms]) OR ("cognitive assess*"[title/abstract]) OR ("cognitive performance" [text word]) OR ("executive function*"[title/abstract]) OR ("neurocognitive performance"[text word]) OR ("cognitive domain*" [title/abstract]))) AND ("cognitive change*"[Title/Abstract] OR "cognitive decline*"[Title/Abstract] OR "cognitive difficult*"[Text Word] OR "cognitive outcome*"[Title/Abstract] OR "neurocognitive dysfunction"[Text Word] OR "longitudinal"[Title/Abstract] OR progress*[title/abstract] OR chronic [title/abstract] OR "cognitive impairment"[title/abstract] OR neurodegen*[text word])

2. Search strategy for EMBASE (Classic + Embase) via Ovid

1. ‘traumatic brain injury’ :ti, ab
2. TBI
3. ‘brain injury’
4. ‘head trauma’
5. exp ‘head injury’/
6. “traumatic brain injury’ :kw
7. 1 OR 2 OR 3 OR 4 OR 5 OR 6
8. exp ‘cognitive ability’/
9. cognition :ab, ti
10. exp ‘cognitive assessment’/
11. ‘cognitive performance’
12. ‘executive function*’
13. exp ‘neurocognitive performance’/
14. ‘cognitive domain*’
15. 8 OR 9 OR 10 OR 11 OR 12 OR 13 OR 14
16. exp ‘cognitive change’/
17. ‘cognitive change*’ :ti, ab, kw
18. exp ‘cognitive decline’/
19. ‘cognitive difficult*’ :ti, ab, kw
20. ‘cognitive outcome*’ :ti, ab, kw
21. ‘cognitive dysfunction’ :ti, ab, kw
22. longitudinal :ab
23. progress* :ti, ab, kw
24. chronic :ab, ti
25. ‘cognitive impairment’ :ti, ab, kw
26. neurodegen* :ti, ab, kw
27. 16 OR 17 OR 18 OR 19 OR 20 OR 21 OR 22 OR 23 OR 24 OR 25 OR 26
28. 7 AND 15 AND 27

Search strategy for APA PsychInfo via EBSCOhost:

A.

1. “traumatic brain injury” AB
2. TBI TX
3. “brain injury” TX
4. “head trauma” TX
5. “brain trauma” TX
6. “head injury” TX
7. “traumatic brain injury” SU

Search: AB "traumatic brain injury" OR TX TBI OR TX "brain injury" OR TX "head trauma" OR TX "brain trauma" OR TX "head injury" OR SU "traumatic brain injury"

B.

1. “cognitive ability” SU
2. cognition SU
3. “cognitive difficult*” TX
4. “cognitive assessment” AB
5. “cognitive performance” AB
6. “executive function*” AB
7. “cognitive domain*” TX

Search: SU "cognitive ability" OR SU cognition OR TX "cognitive difficult*" OR AB "cognitive assessment" OR AB "cognitive performance" OR AB "executive function*" OR TX "cognitive domain*"

C.

1. “cognitive difficult*” TX
2. “cognitive dysfunction” SU
3. “cognitive chang*” AB
4. neurodegener* AB
5. “cognitive outcome*” TX
6. “cognitive decline” TX
7. “cognitive impairment” TX
8. longitudinal AB
9. progress* AB
10. “neurocognitive dysfunction” TX

Search: TX "cognitive difficult*" OR SU "cognitive dysfunction" OR AB "cognitive chang*" OR AB "neurodegener*" OR TX "cognitive outcome*" OR TX "cognitive decline" OR TX "cognitive impairment" OR AB longitudinal OR AB progress* OR TX "neurocognitive dysfunction"

Search: Combine with AND

**Supplementary Table 1**

*Summary table of included studies.*

| **Study** | **Study design** | **Sample size** | **Injury Severity** | **Injury mechanism** | **Timing of assessments** | **Study Main findings** |
| --- | --- | --- | --- | --- | --- | --- |
| Chiou (2019) | Longitudinal comparison between TBI and HC | n = 15 ΤΒΙ,  *M_age_* = 45.80 years (SD = 12.90)*; n = 8 HC | *GCS score not reported,*  PTA > 30 minutes | *Not reported* | 7 years, and 10 years post-injury | Over the study period, TBI patients showed increase in fractional anisotropy values, compared to HC. Increased fractional anisotropy in TBI correlated with improvements in a set-shifting task. |
| Dreer (2008) | Prospective cohort comparison of TBI and HC | n = 24 TBI,  *M_age_* = 30.00 years (SD = 11.70);  n = 20 HC | *M*_GCS_ = 7.1  (SD = 3.2) | *Not reported* | 0, and 6 months post-injury | Impairments in capacity to consent to treatment correlated with short-term verbal memory immediately after the injury. However, at 6-month follow-up language comprehension, executive function and working memory were more strongly correlated with capacity performance. |
| Farbota (2012) | Longitudinal comparison between TBI and HC | n = 20 TBI,  *M*_age_ = 34.50 years, (SD = 12.00);  n = 20 HC | *M*_GCS_ = 5.7  (SD = 4.2) | *Not reported* | 2 months, 10 months, and 4 years post-injury | TBI patients presented with significantly greater total volume loss and annual rate of volume loss, over the study period, compared to HC. Further, they presented with greater atrophy in frontal, temporal, posterior parietal, occipital and inter-hemispheric cortices, relative to HC. Regional volume loss was associated with performance on certain cognitive tasks. |
| Fraser (2019) | Longitudinal comparison between TBI and HC | n = 109 TBI,  *M_age_* = 44.62 years (SD = 16.72);  n = 63 HC | *M*_GCS_ = 9.47 (SD = 4.33),  *M*_PTA_ = 28.17 (SD = 48.95) | Motorvehicle/ traffic accident 69%; falls 13%; work-related injury 5%; assault 2% | 1 month and 4 years post-injury | Significant improvements in cognitive ability observed between baseline and follow‐up. PTA duration was not a significant predictor of cognitive recovery, after controlling for initial cognitive ability. Older age at injury was associated with poorer performance on all cognitive measures at baseline and was a significant predictor of poorer degree of recovery on processing speed, executive function and verbal memory. |
| Hicks (2022) | Prospective controlled comparison of TBI and HC | n = 53 TBI,  *M_age_* = 35.47 years (SD = 14.80);  n = 26 HC | GCS 3-8 = 74.35% *M*_PTA_ = 28.42 (SD = 28.48) | *Not reported* | 0 months, and 12 years post-injury | Trajectory of decline for TBI and HC was similar over 12 years, likely reflecting a healthy ageing process. Age at injury was associated with worse executive outcomes. |
| High (2010) | Double – blind, placebo controlled | n = 11 placebo, *M_age_* = 39.10 years (SD = 8.50)*;  n = 12 experimental | *M*_GCS_ = 6.6  (SD = 3.6) | *Not reported* | 5, and 10 years post-injury | Growth-hormone replacement in deficient TBI patients improved certain neuropsychological measures, compared to the placebo group. Participants receiving growth hormone replacement therapy showed improved hand motor speed, processing speed and verbal learning. |
| Kersel (2001) | Longitudinal follow-up of one cohort | n = 65 TBI,  *M*_age_ = 28 years  (SD = 11) | GCS < 9,  PTA ≥ 24 hours *(exact values not reported)* | Motor vehicle accident n = 49; fall n = 7; assault n = 5; other n = 4 | 6, and 12 months post-injury | At 12 months post-injury impairment was most frequently observed in verbal memory, attention, and executive functioning, with 37% of participants functioning at an impaired level. |
| Macruz (2022) | Longitudinal follow-up of one cohort | n = 25 TBI,  *M_age_* = 28.36 years (SD = 9.17) | GCS 3-8 n = 16; GCS 9-12 n = 9 | Motor vehicle/ traffic accident  n = 24; physical aggression n = 1 | 2, 6, and 12 months post-injury | Patients showed progressive white matter volume and total brain volume loss over the first year following TBI. There was significant improvement in verbal memory, attention, and executive function from 6 to 12 months post-injury. |
| Marsh (2018) | Longitudinal follow-up of one cohort | n = 56 TBI  *M*_age_ = 28.00 years (SD = 12.00) | GCS 3-8 85%; GCS 9-12 13%; GCS 13-15 4% | Motor vehicle/ traffic accident  n = 40; fall n = 7; assault n = 5; other n = 4 | 6 months, 12 months, and 5 years post-injury | Compared to normative data, TBI patients showed improvement on immediate memory and total learning in the first year. Executive functioning overall improved over time. |
| Millis (2001) | Longitudinal follow-up of one cohort | n = 182 TBI  *M*_age_ = 39.90 years (SD = 14.30)* | GCS 3-8 22%; GCS 9-12 12%; GCS 13-15 20%; GCS unavailable (due to intubation) 45% | *Not reported* | 12 months, and 5 years post-injury | Five years post-injury, cognitive impairments were more likely in memory, attention, and processing speed. However, some patients showed improvement in attention, cognitive speed, visuospatial ability, verbal fluency, and memory from 1 to 5 years post-injury. Cognitive decline was observed in 15.2% of the sample. |
| Novack (2000) | Prospective longitudinal follow-up of one cohort | n = 72 TBI  *M*_age_ = 31.10 years (range 16-70 years) | GCS 3-8 68%; PTA ≥ 7 83.1% | Motor vehicle accident 65%; firearm/ other assaults 20%; falls 11% | 6, and 12 months post-injury | Patients showed significant overall improvement from 6 to 12 months post-injury, with the largest improvements noted on memory measures. At 12 months post-injury, those with severe TBI were still impaired, whereas those with moderate TBI functioned at a low-average level. |
| Rabinowitz (2017) | Prospective cohort comparison of TBI and HC | n = 46 TBI  *M*_age_ = 35.20 years, (SD = 15.00);  n = 38 HC | *M*_GCS_ = 9.5  (SD = 4.2),  *M*_PTA_ = 26.5  (SD = 21.2) | Motor vehicle accident 70%; falls 20%; intentional injury 10% | 3, 6, and 12 months post-injury | Verbal learning and executive functions showed linear improvement over time among TBI patients. Processing speed recovery followed a curvilinear trajectory of improvement. Although education was associated with initial level of functioning, it was not a significant factor in the recovery trajectory. The extent of diffuse axonal injury influenced initial processing speed and executive functioning but not rate of recovery. |
| Schultz (2021) | Longitudinal follow-up comparison of TBI and HC | n = 23 TBI  *M*_age_ = 33.19 years (range 20-56);  n = 23 HC | *M*_PTA_ = 51.52  (range 9 – 128) | Motor vehicle accident n = 11; falls n = 6; assault n = 2; other n = 2 | 3 months, and then monthly for the ﬁrst 12 months post-injury | Monthly assessments revealed a typically continuous trajectory of cognitive recovery. Four distinct patterns of cognitive recovery were observed depending on the cognitive domain: (1) no change; (2) slow recovery; (3) steep linear recovery; (4) steeper recovery. Despite improvements, a significant portion of participants remained cognitively impaired at 12 months post-injury. |
| Sigurdardottir (2020) | Prospective longitudinal follow-up of one cohort | n = 79 TBI  *M*_age_ = 30.20 years, (SD = 10.50) | *M*_GCS_ = 7.8  (SD = 3.1),  *M*_PTA_ = 24.2  (SD = 29.6) | *Not reported* | 3 months, 12 months, 5 years, and 10 years post-injury | There were significant improvements on memory, executive function, and reasoning over time, with the largest gains observed over the first year post-injury. A portion of participants (11%) showed significant decline, particularly in memory measures. Worse memory performance was associated with PTA greater than 3 weeks. |
| Slavoaca (2020) | Prospective cohort follow-up | n = 62 TBI  *M*_age_ = 38.00 years, (SD = 18.00) | *M*_GCS_ = 10.40 (SD = 1.40) | *Not reported* | 0, and 3 months post-injury | Serum levels of Neuron-Specific Enolase (NSE) four hours after the injury correlated with cognitive outcomes at 10 and 90 days post-injury. Serum S100 did not correlate with cognitive outcomes. Findings suggest that NSE may be used as an early biomarker to detect patients at risk of poorer cognitive outcomes. |
| Spitz (2012) | Longitudinal follow-up of one cohort | n = 111 TBI,  *M*_age_ = 32.32 years, (SD = 13.33);  n = 79 HC | *M*_PTA_ = 28.69 (SD = 29.47) | *Not reported* | 3, and 13 months post-injury. | Patients showed some improvement on cognitive and functional outcomes from 3 to 13 months post-injury. However, they remained significantly impaired compared to HC in memory, processing speed, and executive functioning. Lower cognitive ability was associated with greater functional difficulties. |
| Stenberg (2015) | Prospective multicentre of a TBI cohort | n = 114 TBI, *Median*_age_ = 42 years  (range 17-65) | *Median*_GCS_ = 5 (range 3-8) | Motor vehicle accident n = 46; falls n = 50; other n = 13; Missing n = 5 | 0, 3, and 12 months post-injury | Higher performance on orientation, visuospatial, visual problem solving, memory, affect and awareness subscales of the Barrow Neurological Institute Screen for Higher Cerebral Functions (BNIS) at 3 months post-injury, were associated with better scores one-year post-injury. Significant improvements in cognitive ability were only observed from 3 weeks to 3 months post-injury. |
| Tabet (2021) | Longitudinal comparison of TBI and orthopaedic controls | n = 44 mild TBI;  n = 31 moderate-severe TBI,  *M*_age_ = 52.29 years (SD = 19.39);  n = 50 orthopaedic controls | GCS < 13,  PTA ≥ 24 hours *(exact values not reported)* | Fall n = 21; motor vehicle accident n = 5; assault n = 1; other n = 3 | 1, and 6 months post-injury | There was significant improvement of TBI patients in verbal cognitive switching from baseline to follow-up, relative to orthopaedic controls. All groups improved significantly on the backward digit span over time. |
| Vijayakumari (2021) | Cross-sectional with follow-up comparison of TBI and HC | n = 24 TBI,  *M_age_* = 34.97 years (SD = 15.22);  n = 20 HC | *M*_GCS_ = 9.76 (SD = 4.48),  *M*_PTA_ = 27.50 (SD = 4.48) | *Not reported* | 3,6, and 12 months post-injury | There was widespread increase in free water volume fraction 3 months after the injury. Patient-specific summary scores of free water volume fraction may serve as biomarkers for injury severity and functional and cognitive outcomes in moderate-severe TBI. |
| Ylioja (2010)** | Longitudinal comparison of penetrating and non-penetrating TBI | n = 61 penetrating TBI, *M*_age_ = 27.57 years (SD = 10.20);  n = 61 non-penetrating TBI, *M*_age_ = 29.97 years (SD = 13.89) | PTA ≥ 7 days 85.2% penetrating group, 88.5% non-penetrating group | Gunshot wound n = 61; motor vehicle accident n = 61 | 0 months, 12 months, and 2 years post-injury | The penetrating group had poorer attention immediately after the injury, but the two groups were similar in subsequent time points. Verbal learning was superior in the penetrating group at baseline but was not significantly different between the two groups by year 1. Overall, the penetrating group showed greater variability in cognitive outcomes. |
| Zaninotto (2017) | Prospective longitudinal follow-up of one cohort | n = 40 TBI,  *M*_age_ = 28.7 years (SD = 9.4) | GCS < 13 with Diffuse Axonal Injury, 60% severe TBI | Motor vehicle accident n = 32; running over n = 6; other n = 2 | 6, and 12 months post-injury | Participants showed significant improvement in their visuospatial memory from baseline to follow-up. Lower baseline visuospatial ability was associated with greater improvement at follow-up. |

*Note.* Reported age refers to age at injury, unless otherwise stated; TBI = Traumatic Brain Injury; SD = Standard Deviation; HC = Healthy Controls; GCS = Glasgow Coma Scale; PTA = Post Traumatic Amnesia in days, unless otherwise stated. *Age at study entry; **Results were combined into one group for meta-analysis

**Supplementary Table 2**

*Risk of bias (RoB) for quantitative, cohort studies evaluated with the Newcastle–Ottawa*

*Scale*

| **Study** | **Selection (0-4)** | **Comparability (0-2)** | **Outcome (0-3)** |
| --- | --- | --- | --- |
| Chiou (2019) | ★★★ | ★★ | ★★ |
| Dreer (2008) | ★★★ | ★★ | ★★ |
| Farbota (2012) | ★★★★ | ★★ | ★★★ |
| Feltrin (2018) | ★★★ | ★★ | ★★★ |
| Fraser (2019) | ★★★ | ★★ | ★★★ |
| Kersel (2001) | ★★★ | ★★ | ★★★ |
| Macruz (2022) | ★★★ | ★★ | ★★★ |
| Marsh (2018) | ★★★ | ★★ | ★★★ |
| Mills (2001) | ★★★ | ★★ | ★★ |
| Novack (2000) | ★★ | ★★ | ★★★ |
| Rabinowitz (2017) | ★★★ | ★★ | ★★ |
| Schultz (2021) | ★★★★ | ★★ | ★★★ |
| Sigurdardottir (2020) | ★★★ | ★★ | ★★★ |
| Slavoaca (2020) | ★★ | ★★ | ★★★ |
| Spitz (2012) | ★★★ | ★★ | ★★★ |
| Stenberg (2015) | ★★★ | ★★ | ★★ |
| Tabet (2024) | ★★★★ | ★★ | ★★★ |
| Vijayakumari (2021) | ★★★ | ★★ | ★★ |
| Ylioja (2010) | ★★ | ★★ | ★★ |
| Zaninotto (2017) | ★★★ | ★★ | ★★★ |

*Note.* A greater number of stars indicates greater study quality for each domain (selection, comparability, outcome). Studies with six or more stars are suggested to be high quality.

**Supplementary Table 3**

*Risk of bias (RoB) for quantitative, case control studies evaluated with the Newcastle–Ottawa Scale*

| **Study** | **Selection (0-4)** | **Comparability (0-2)** | **Outcome (0-3)** |
| --- | --- | --- | --- |
| Hicks (2022) | ★★★★ | ★★ | ★★ |

*Note.* A greater number of stars indicates greater study quality for each domain (selection, comparability, outcome). Studies with six or more stars are suggested to be high quality.

**Supplementary Table 4**

*Risk of bias in individual randomised studies, based on the Critical Appraisals Skills Programme (CASP) checklist for randomised controlled trials*

| **Domain** | **High (2010)** |
| --- | --- |
| **Did the trial address a clearly focused issue?** | **+** |
| **Was the assignment of patients randomised?** | **+** |
| **Were all participants who entered the study accounted for at its conclusion?** | **?** |
| **Were the patients, health workers and study personnel ‘blind’ to treatment?** | **+** |
| **Were the groups similar at the start of the trial?** | **-** |
| **Aside from experimental intervention, were the groups treated equally?** | **?** |
| **Can the results be applied in your local population or context?** | **+** |
| **Were all clinically important outcomes considered?** | **+** |
| **Are the benefits worth the harm and costs?** | **+** |

*Note.* The above domains were assessed for each study and risk of bias was judged as high (-), unclear (?) or low (+).

**Supplementary Table 5**

*Cognitive tests used to assess memory ability across included studies*

| **Main cognitive ability assessed** | **Instrument and variables** | **Evidence for cognitive domain** | **Study** |
| --- | --- | --- | --- |
| *Attention/ auditory working memory* | Digit Span (WAIS* III; forward/ backward; total recalled) (Wechsler, 1977) | Lezak et al., 2012 | Dreer (2008); Farbota (2012); Hicks (2022); High (2010); Kersel (2001); Marsh (2018); Millis (2001); Novack (2000); Rabinowitz (2017); Schultz (2022); Tabet (2024); Zaninotto (2017) |
| *Auditory memory* | Logical Memory I, II (total recalled) (Wechsler, 1977) | Lezak et al., 2012 | Dreer (2008); High (2010); Millis (2001) |
| *Verbal memory* | Rey Auditory Verbal Learning Task (RAVLT; total recalled) (Rey, 1983) | Lezak et al., 2012 | Dreer (2008); Fraser (2019); Hicks (2022); Marsh (2018); Millis (2001); Rabinowitz (2017); Spitz (2012); Vijaykumari (2021); Ylioja (2010) |
| *Verbal memory* | California Verbal Learning Task (CVLT; immediate/ delayed; total recalled) (Delis et al., 1987) | Delis et al., 1988 | Novack (2000); Sigurdardottir (2020); Ylioja (2010) |
| *Verbal memory* | Hopkins Verbal Learning Task (HVLT; immediate/ delayed; total recalled) (Brandt, 1991) | Rasmusson et al., 1995 | Feltrin (2018); Macruz (2022); Schultz (2022) |
| *Verbal, visual, auditory memory* | Working Memory Index (WAIS* III; total score) (Wechsler, 1977) | Lezak et al., 2012 | High (2010); Spitz (2012) |
| *Visual memory* | Rey Osterrieth Complex Figure (ROCF; recall trial; total score) (Rey & Osterrieth, 1993) | Zhang et al., 2021 | High (2010); Macruz (2022); Sigurdardottir (2020); Zaninotto (2017) |
| *Attention/ auditory working memory* | Letter number sequencing (WAIS* III; longest correctly recalled trial) (Wechsler, 1977) | Lezak et al., 2012 | Rabinowitz (2017); Sigurdardottir (2020) |
| *Auditory memory, verbal memory* | Barrow Neurological Institute Screen (BNIS; memory domain; total score) (Prigatano et al., 1995) | Boosman et al., 2013 | Stenberg (2015) |

*Note*. * Weschler Adult Intelligence Scale. Only raw scores were considered for each test, except for WAIS test results, which are always standardised.

**Supplementary Table 6**

*Cognitive tests used to assess processing speed across included studies*

| **Main cognitive ability assessed** | **Instrument and variables** | **Evidence for cognitive domain** | **Study Author** |
| --- | --- | --- | --- |
| *Rapid visual scanning* | Symbol Modalities Digit Task (SMDT; number of correct matches) (Smith, 1973) | Lezak et al., 2012 | Dreer (2008); Millis (2001); Schultz (2022); Spitz (2012); Ylioja (2010) |
| *Rapid visual scanning* | Trail Making Test A (TMT-A; completion time) (Reitan, 1956) | Schinka et al., 2010; Lezak et al., 2012 | Dreer (2008); Farbota (2012); Feltrin (2018); High (2010); Macruz (2022); Millis (2001); Novack (2000); Spitz (2012); Ylioja (2010) |
| *Rapid visual scanning* | Digit Symbol Coding Task (DCST; number of correct matches) (WAIS* III) (Wechsler, 1977) | Lezak et al., 2012 | Fraser (2019); Hicks (2022); High (2010); Marsh (2022); Kersel (2001); Slavoaca (2020) |
| *Processing speed* | Processing Speed Index (WAIS* III; total score) (Wechsler, 1977) | Delis et al., 2001 | High (2010); Rabinowitz (2017); Vijaykumari (2021) |
| *Rapid visual scanning* | Symbol search (WAIS* III; number of correct matches) (Wechsler, 1977) | Sweet, 2011 | High (2010); Slavoaca (2020) |

*Note*. * Weschler Adult Intelligence Scale. Only raw scores were considered for each test, except for WAIS test results, which are always standardised.

**Supplementary Table 7**

*Cognitive tests used to assess executive functioning across included studies*

| **Main cognitive ability assessed** | **Instrument and variables** | **Evidence for cognitive domain** | **Study** |
| --- | --- | --- | --- |
| *Cognitive switching* | Trail Making Test B (TMT-B; completion time) (Reitan, 1956) | Kortte et al., 2002;  Lezak et al., 2012 | Chiou (2019); Dreer (2008); Farbota (2012); Feltrin (2018); Fraser (2019); Hicks (2022); High (2010); Macruz (2022); Millis (2001); Rabinowitz (2018); Sigurdardottir (2020); Spitz (2012); Ylioja (2010) |
| *Inhibitory control, motor programming, initiation behaviour* | Executive Interview (EXIT-25; total score) (Royall et al., 1992) | Moreira et al., 2017 | Dreer (2008) |
| *Response inhibition* | Stroop Test (interference trial; total correct) (Stroop, 1935) | Schinka et al., 2010;  Lezak et al., 2012 | Schultz (2022); Slavoaca (2020) |
| *Response inhibition* | Color-Word Interference Test (CWIT; inhibition/ switching trials; total score) (Delis et al., 2001) | Lezak et al., 2012 | Chiou (2019); Sigurdardottir (2020); Tabet (2024) |

**Supplementary Table 8**

*Cognitive tests used to assess verbal ability across included studies*

| **Main cognitive ability assessed** | **Instrument and variables** | **Evidence for cognitive domain** | **Study Author** |
| --- | --- | --- | --- |
| *Word fluency* | Control Oral Word Association Test (COWAT) (Benton et al., 1983) | Schinka et al., 2010; Lezak et al., 2012 | Dreer (2008); Farbota (2012); High (2010); Kersel (2001); Marsh (2022); Millis (2001); Novack (2000); Rabinowitz (2017); Spitz (2012) |
| *Verbal ability* | Similarities (WAIS* III; total correct) (Wechsler, 1977) | Lezak et al., 2012 | High (2010); Kersel (2001); Novack (2000); Schultz (2022); Sigurdardottir (2020); Slavoaca (2020) |
| *Confrontation naming* | Hundred Pictures Naming Test (HPNT; total correct) (Fisher & Glenister, 1992) | Fisher & Glenister, 1992 | Schultz (2022) |
| *Verbal knowledge* | Vocabulary (WAIS* III; total correct) (Wechsler, 1977) | Lezak et al., 2012 | High (2010); Novack (2000) |
| *Verbal comprehension* | Token Test (total correct) (De Renzi & Faglioni, 1978) | Lezak et al., 2012 | Dreer (2008); High (2010); Millis (2001); Schultz (2022) |
| *Verbal fluency* | Delis-Kaplan Executive Function system (DKEFS-verbal fluency; total score) (Delis et al., 2001) | Strong et al., 2010 | Sigurdardottir (2020) |
| *Verbal generation, comprehension, fluency* | Barrow Neurological Institute Screen (BNIS-language domain; total score) (Prigatano et al., 1995) | Boosman et al., 2013 | Stenberg (2015) |
| *Verbal generation* | Verbal generativity (animal/ vegetable/ clothing; number of words generated) (Lezak et al., 2004) | Lezak et al., 2012 | Dreer (2008) |
| *Verbal generation* | Letter fluency (number of words generated) (Schultz et al., 2022) | Ruff et al., 1996 | Schultz (2022) |

*Note*. * Weschler Adult Intelligence Scale. Only raw scores were considered for each test, except for WAIS test results, which are always standardised.

**Supplementary Table 9**

*Cognitive tests used to assess visuospatial ability across included studies*

| **Main cognitive ability assessed** | **Instrument and variables** | **Evidence for cognitive domain** | **Study Author** |
| --- | --- | --- | --- |
| *Visuospatial conceptualisation* | Block design (WAIS* III; total score) (Wechsler, 1977) | Lezak et al., 2012 | Dreer (2008); High (2010); Kersel (2001); Marsh (2022); Millis (2001); Novack (2000) |
| *Perceptual organisation* | Rey Osterrieth Complex Figure (ROCF-copy; score) (Rey & Osterrieth, 1993) | Lezak et al., 2012 | High (2010); Zaninotto (2017); Macruz (2022) |
| *Visual recognition* | Judgement of Line Orientation Test (JLOT; total correct) | Lezak et al., 2012 | Schultz (2022) |
| *Perceptual organisation* | Medical College of Georgia Complex Figure Test (total score) (Loring & Meador, 2003) | Lezak et al., 2012 | Schultz (2022) |
| *Perceptual organisation* | Barrow Neurological Institute Screen (BNIS-visuospatial domain; total score) (Prigatano et al., 1995) | Boosman et al., 2013 | Stenberg (2015) |

*Note*. * Weschler Adult Intelligence Scale. Only raw scores were considered for each test, except for WAIS test results, which are always standardised.


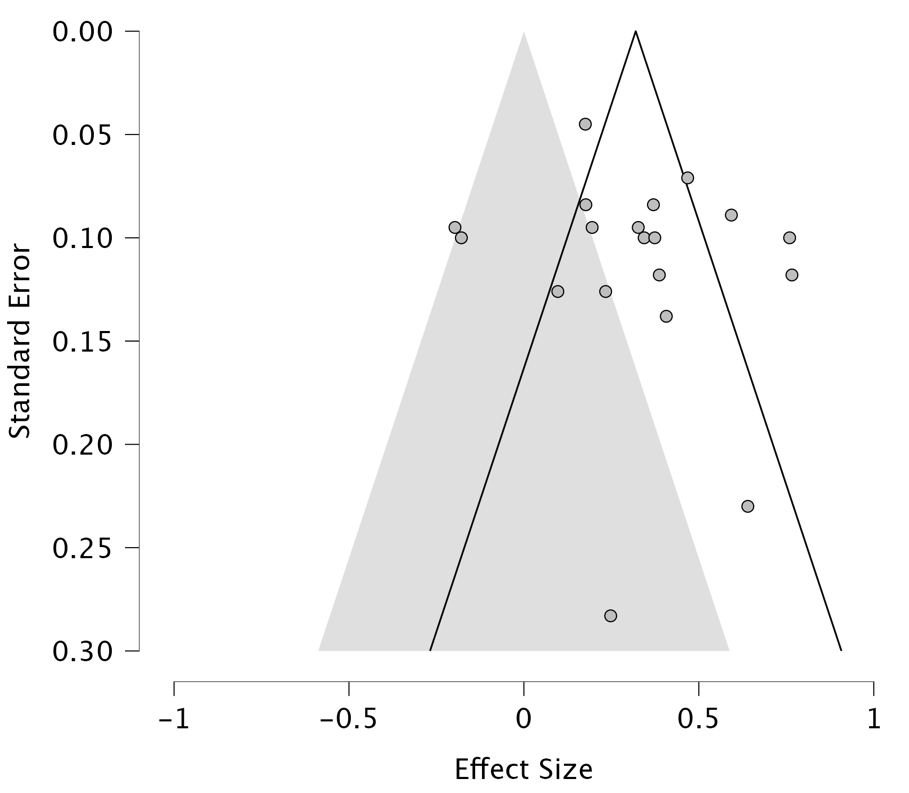


**Supplementary Fig. 1.** Funnel plot of the meta-analysis of longitudinal changes in memory following moderate-severe TBI. Each plotted point represents the standard error and effect size between baseline and follow-up memory assessment in a single study. The shaded triangle represents the region where 95% of the data points would lie in the absence of publication bias, under the null hypothesis. The straight line represents the region where 95% of the data points would lie under a Random Effects Maximum Likelihood Estimator (REML), which aligns with the alternative hypothesis.


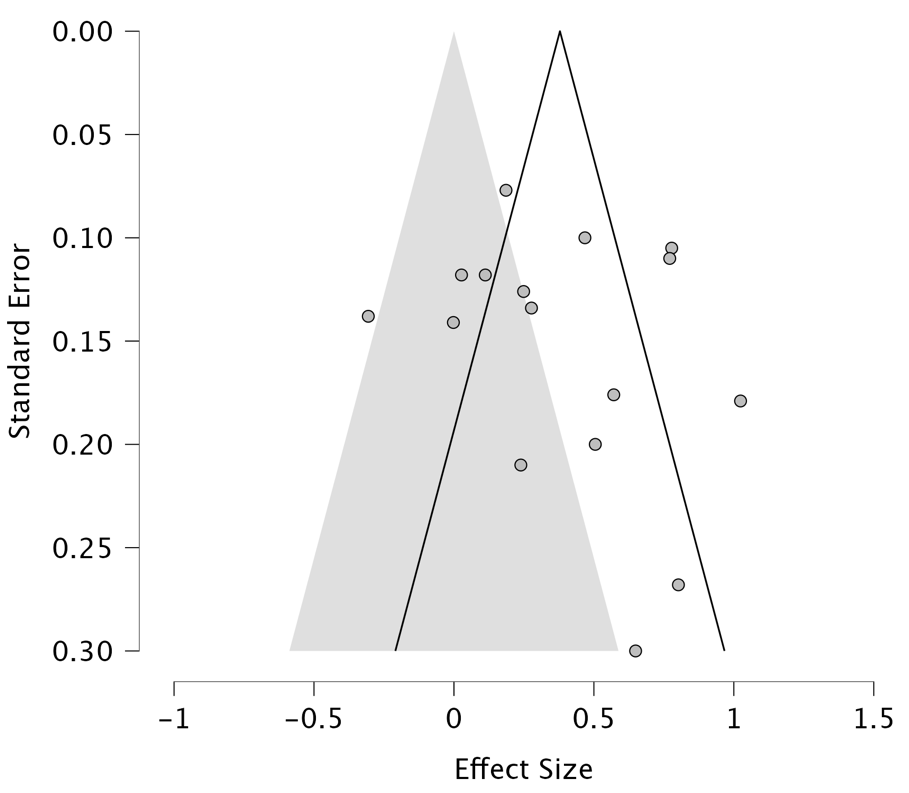


**Supplementary Fig. 2.** Funnel plot of the meta-analysis of longitudinal changes in processing speed following moderate-severe TBI. Each plotted point represents the standard error and effect size between baseline and follow-up processing speed assessment in a single study. The shaded triangle represents the region where 95% of the data points would lie in the absence of publication bias, under the null hypothesis. The straight line represents the region where 95% of the data points would lie under a Random Effects Maximum Likelihood Estimator (REML), which aligns with the alternative hypothesis.


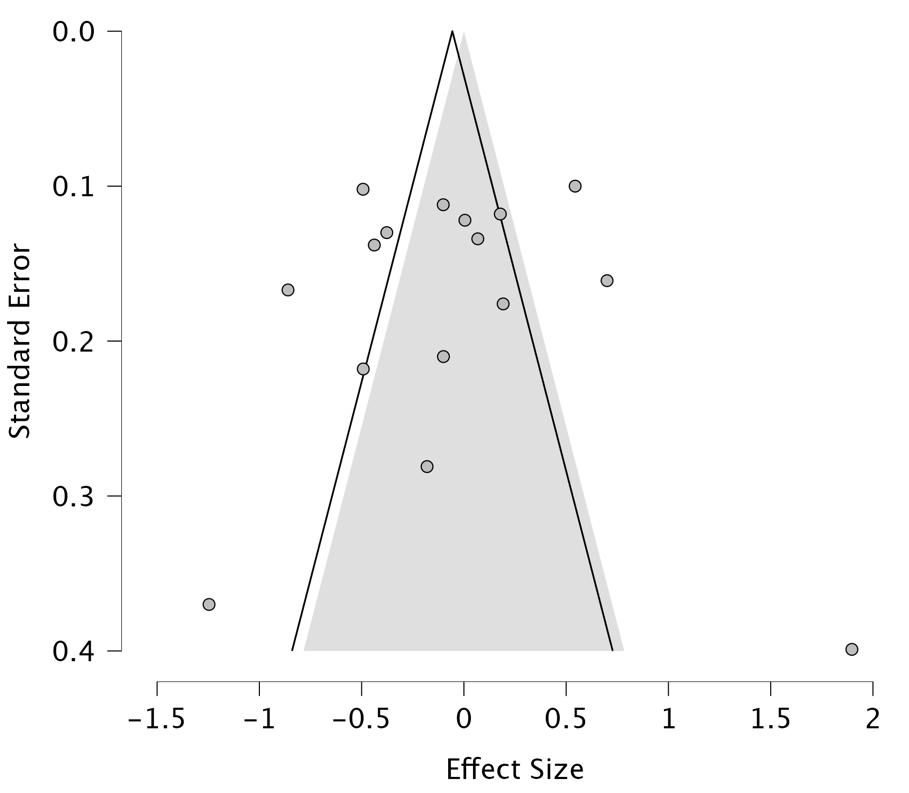


**Supplementary Fig. 3.** Funnel plot of the meta-analysis of longitudinal changes in executive functioning following moderate-severe TBI. Each plotted point represents the standard error and effect size between baseline and follow-up executive functioning assessment in a single study. The shaded triangle represents the region where 95% of the data points would lie in the absence of publication bias, under the null hypothesis. The straight line represents the region where 95% of the data points would lie under a Random Effects Maximum Likelihood Estimator (REML), which aligns with the alternative hypothesis.


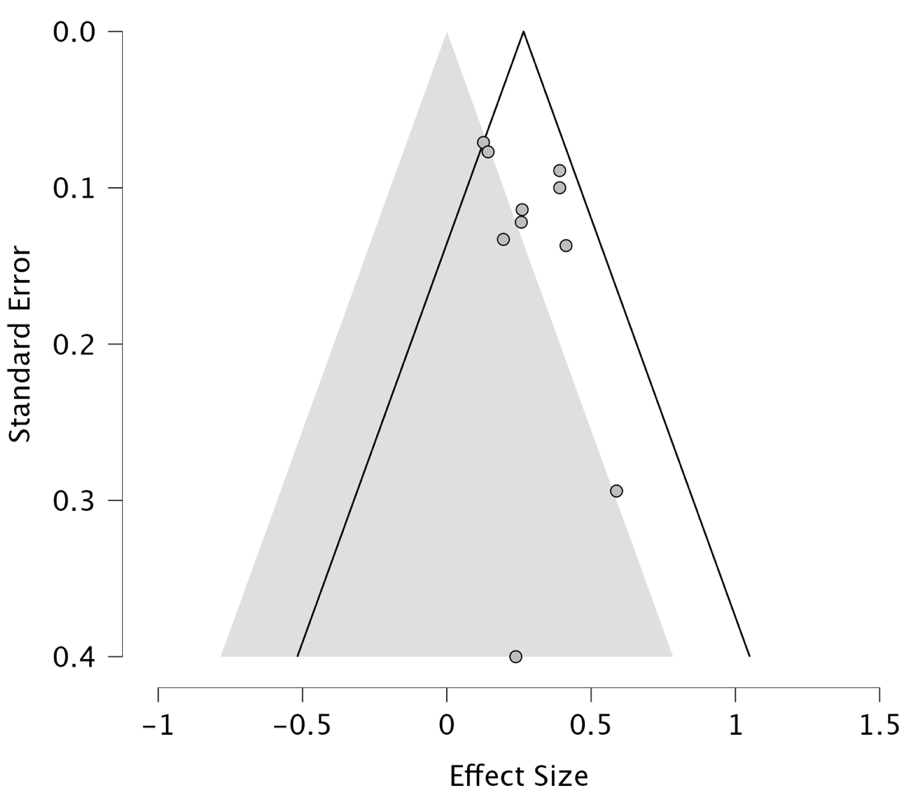


**Supplementary Fig. 4.** Funnel plot of the meta-analysis of longitudinal changes in verbal ability following moderate-severe TBI. Each plotted point represents the standard error and effect size between baseline and follow-up verbal ability assessment in a single study. The shaded triangle represents the region where 95% of the data points would lie in the absence of publication bias, under the null hypothesis. The straight line represents the region where 95% of the data points would lie under a Random Effects Maximum Likelihood Estimator (REML), which aligns with the alternative hypothesis.


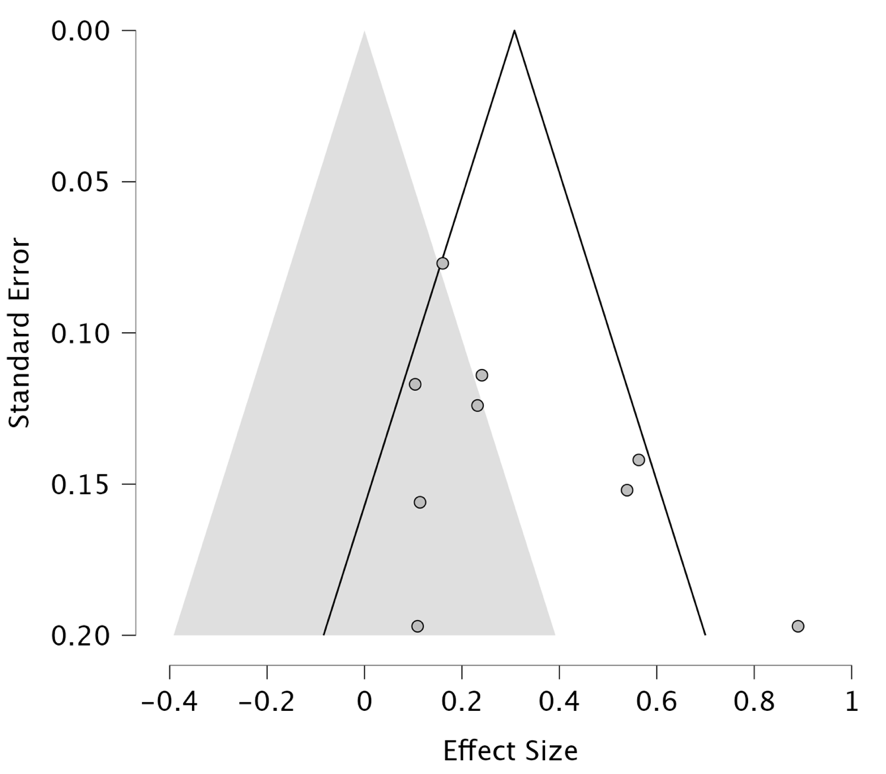


**Supplementary Fig. 5.** Funnel plot of the meta-analysis of longitudinal changes in visuospatial ability following moderate-severe TBI. Each plotted point represents the standard error and effect size between baseline and follow-up visuospatial ability assessment in a single study. The shaded triangle represents the region where 95% of the data points would lie in the absence of publication bias, under the null hypothesis. The straight line represents the region where 95% of the data points would lie under a Random Effects Maximum Likelihood Estimator (REML), which aligns with the alternative hypothesis.

**Supplementary References**

Benton, A. L., Hamsher, de S. K., & Sivan, A. B. (1983). *Controlled Oral Word Association Test (COWAT)*. https://doi.apa.org/doi/10.1037/t10132-000

Boosman, H., Visser-Meily, J. M. A., Post, M. W. M., Duits, A., & Van Heugten, C. M. (2013). Validity of the Barrow Neurological Institute (BNI) Screen for Higher Cerebral Functions in Stroke Patients with Good Functional Outcome. *The Clinical Neuropsychologist*, *27*(4), 667–680. https://doi.org/10.1080/13854046.2013.777787

Brandt, J. (1991). The Hopkins Verbal Learning Test: Development of a new memory test with six equivalent forms. *Clinical Neuropsychologist*, *5*(2), 125–142. https://doi.org/10.1080/13854049108403297

Chiou, K. S., Jiang, T., Chiaravalloti, N., Hoptman, M. J., DeLuca, J., & Genova, H. (2019). Longitudinal examination of the relationship between changes in white matter organization and cognitive outcome in chronic TBI. *Brain Injury*, *33*(7), 846–853. https://doi.org/10.1080/02699052.2019.1606449

De Renzi, E., & Faglioni, P. (1978). Normative data and screening power of a shortened version of the Token Test. *Cortex*, *14*(1), 41–49. https://doi.org/10.1016/s0010-9452(78)80006-9

Delis, D. C., Freeland, J., Kramer, J. H., & Kaplan, E. (1988). Integrating clinical assessment with cognitive neuroscience: Construct validation of the California Verbal Learning Test. *Journal of Consulting and Clinical Psychology*, *56*(1), 123–130. https://doi.org/10.1037/0022-006X.56.1.123

Delis, D. C., Kaplan, F., & Kramer, J. H. (2001). *Delis-Kaplan executive function system*. The Psychological Corporation.

Delis, D. C., Kramer, J. H., Kaplan, E., & Ober, B. A. (1987). California Verbal Learning Test. *California Verbal Learning Test (CVLT)*. https://doi.org/10.1037/t15072-000

Dreer, L. E., DeVivo, M. J., Novack, T. A., Krzywanski, S., & Marson, D. C. (2008). Cognitive predictors of medical decision-making capacity in traumatic brain injury. *Rehabilitation Psychology*, *53*(4), 486–497. https://doi.org/10.1037/a0013798

Farbota, K. D. M., Sodhi, A., Bendlin, B. B., McLaren, D. G., Xu, G., Rowley, H. A., & Johnson, S. C. (2012). Longitudinal Volumetric Changes following Traumatic Brain Injury: A Tensor-Based Morphometry Study. *Journal of the International Neuropsychological Society*, *18*(6), 1006–1018. https://doi.org/10.1017/S1355617712000835

Fisher, J. P., & Glenister, J. M. (1992). *The hundred pictures naming test manual*. Australian Council for Educational Research.

Fraser, E. E., Downing, M. G., Biernacki, K., McKenzie, D. P., & Ponsford, J. L. (2019). Cognitive Reserve and Age Predict Cognitive Recovery after Mild to Severe Traumatic Brain Injury. *Journal of Neurotrauma*, *36*(19), 2753–2761. https://doi.org/10.1089/neu.2019.6430

Hicks, A. J., Spitz, G., Rowe, C. C., Roberts, C. M., McKenzie, D. P., & Ponsford, J. L. (2022). Does cognitive decline occur decades after moderate to severe traumatic brain injury? A prospective controlled study. *Neuropsychological Rehabilitation*, *32*(7), 1530–1549. https://doi.org/10.1080/09602011.2021.1914674

High, W. M., Briones-Galang, M., Clark, J. A., Gilkison, C., Mossberg, K. A., Zgaljardic, D. J., Masel, B. E., & Urban, R. J. (2010). Effect of Growth Hormone Replacement Therapy on Cognition after Traumatic Brain Injury. *Journal of Neurotrauma*, *27*(9), 1565–1575. https://doi.org/10.1089/neu.2009.1253

Kortte, K. B., Horner, M. D., & Windham, W. K. (2002). The trail making test, part B: Cognitive flexibility or ability to maintain set? *Applied Neuropsychology*, *9*(2), 106–109. https://doi.org/10.1207/S15324826AN0902_5

Lezak, M. D., Howieson, D. B., Bigler, E. D., & Tranel, D. (2012). *Neuropsychological assessment* (Fifth edition). Oxford University Press.

Loring, D. W., & Meador, K. J. (2003). The Medical College of Georgia (MCG) complex figures: Four gorms for follow-up. In *The handbook of Rey-Osterrieth complex figure usage: Clinical and research applications*. Psychological Assessment Resources.

Macruz, F. B. D. C., Feltrin, F. S., Zaninotto, A., Guirado, V. M. D. P., Otaduy, M. C. G., Tsunemi, M. H., Nucci, M. P., Rimkus, C., Andrade, C. S., & Leite, C. D. C. (2022). Longitudinal assessment of magnetization transfer ratio, brain volume, and cognitive functions in diffuse axonal injury. *Brain and Behavior*, *12*, e2490. https://doi.org/10.1002/brb3.2490

Millis, S. R., Rosenthal, M., Novack, T. A., Sherer, M., Nick, T. G., Kreutzer, J. S., High, W. M., & Ricker, J. H. (2001). Long-Term Neuropsychological Outcome After Traumatic Brain Injury: *The Journal of Head Trauma Rehabilitation*, *16*(4), 343–355. https://doi.org/10.1097/00001199-200108000-00005

Moreira, H. S., Costa, A. S., Castro, S. L., Lima, C. F., & Vicente, S. G. (2017). Assessing Executive Dysfunction in Neurodegenerative Disorders: A Critical Review of Brief Neuropsychological Tools. *Frontiers in Aging Neuroscience*, *9*, 369. https://doi.org/10.3389/fnagi.2017.00369

Prigatano, G. P., Amin, K., & Rosenstein, L. D. (1995). *Administration and scoring manual for the BNI Screen for higher cerebral functions*. Barrow Neurological Institute.

Rabinowitz, A. R., Hart, T., Whyte, J., & Kim, J. (2018). Neuropsychological Recovery Trajectories in Moderate to Severe Traumatic Brain Injury: Influence of Patient Characteristics and Diffuse Axonal Injury. *Journal of the International Neuropsychological Society*, *24*(3), 237–246. https://doi.org/10.1017/S1355617717000996

Rasmusson, D. X., Bylsma, F. W., & Brandt, J. (1995). Stability of performance on the Hopkins Verbal Learning Test. *Archives of Clinical Neuropsychology*, *10*(1), 21–26. https://doi.org/10.1016/0887-6177(94)E0001-6

Reitan, R. M. (1956). *Trail Making test. Manual for administration, scoring and interpretation*. University Press.

Rey, A. (1983). *Rey Auditory-Verbal Learning Test*.

Rey, A., & Osterrieth, P. A. (1993). *Translations of excerpts from Rey’s ‘Psychological Examination of Traumatic Encephalopathy’ and PA Osterrieth’s The complex figure copy test*. *7*, 2–21.

Royall, D. R., Mahurin, R. K., & Gray, K. F. (1992). Bedside Assessment of Executive Cognitive Impairment: The Executive Interview. *Journal of the American Geriatrics Society*, *40*(12), 1221–1226. https://doi.org/10.1111/j.1532-5415.1992.tb03646.x

Ruff, R. M., Light, R. H., Parker, S. B., & Levin, H. S. (1996). Benton controlled oral word association test: Reliability and updated norms. *Archives of Clinical Neuropsychology*, *11*(4), 329–338. https://doi.org/10.1093/arclin/11.4.329

Schinka, J. A., Loewenstein, D. A., Raj, A., Schoenberg, M. R., Banko, J. L., Potter, H., & Duara, R. (2010). Defining Mild Cognitive Impairment: Impact of Varying Decision Criteria on Neuropsychological Diagnostic Frequencies and Correlates. *The American Journal of Geriatric Psychiatry*, *18*(8), 684–691. https://doi.org/10.1097/JGP.0b013e3181e56d5a

Schultz, R., Tate, R. L., & Perdices, M. (2022). Neuropsychological recovery during the first 12 months after severe traumatic brain injury: A longitudinal study with monthly assessments. *Neuropsychological Rehabilitation*, *32*(7), 1291–1323. https://doi.org/10.1080/09602011.2021.1882507

Sigurdardottir, S., Andelic, N., Røe, C., & Schanke, A. K. (2020). Trajectory of 10-Year Neurocognitive Functioning After Moderate–Severe Traumatic Brain Injury: Early Associations and Clinical Application. *Journal of the International Neuropsychological Society*, *26*(7), 654–667. https://doi.org/10.1017/S1355617720000193

Slavoaca, D., Birle, C., Stan, A., Tatomir, A., Popa, O., Rosu, P., Vulcan, A. M., Chira, D., Popa, L. L., Dina, C., Vacaras, V., Strilciuc, S., & Vos, P. (2020). Prediction of Neurocognitive Outcome after Moderate-Severe Traumatic Brain Injury Using Serum Neuron-Specific Enolase and S100 biomarkers. *Journal of Medicine and Life*, *13*(3), 306–313. https://doi.org/10.25122/jml-2020-0147

Smith, A. (1973). *Symbol Digit Modalities Test (SDMT)*. Western Psychological Services.

Spitz, G., Ponsford, J. L., Rudzki, D., & Maller, J. J. (2012). Association between cognitive performance and functional outcome following traumatic brain injury: A longitudinal multilevel examination. *Neuropsychology*, *26*(5), 604–612. https://doi.org/10.1037/a0029239

Strong, C. A. H., Tiesma, D., & Donders, J. (2010). Criterion Validity of the Delis-Kaplan Executive Function System (D-KEFS) Fluency Subtests After Traumatic Brain Injury. *Journal of the International Neuropsychological Society*, *17*(2), 230–237. https://doi.org/10.1017/S1355617710001451

Stroop, J. R. (1935). Studies of interference in serial verbal reactions. *Journal of Experimental Psychology*, *18*(6), 643–662. https://doi.org/10.1037/h0054651

Sweet, L. H. (2011). Symbol Search. In J. S. Kreutzer, J. DeLuca, & B. Caplan (Eds.), *Encyclopedia of Clinical Neuropsychology* (pp. 2444–2445). Springer. https://doi.org/10.1007/978-0-387-79948-3_1336

Tabet, S., Laguë-Beauvais, M., Francoeur, C., Sheehan, A., Abouassaly, M., Marcoux, J., Dagher, J. H., Ursulet, A., Colucci, E., & De Guise, E. (2024). Longitudinal recovery of executive functions and social participation prediction following traumatic brain injury. *Applied Neuropsychology: Adult*, *31*(2), 134–143. https://doi.org/10.1080/23279095.2021.2002866

Wechsler, D. (1977). *Wechsler Adult Intelligence Scale* (3rd ed.). The Psychological Corporation.

Ylioja, S., Hanks, R., Baird, A., & Millis, S. (2010). Are Cognitive Outcome and Recovery Different in Civilian Penetrating Versus Non-Penetrating Brain Injuries? *The Clinical Neuropsychologist*, *24*(7), 1097–1112. https://doi.org/10.1080/13854046.2010.516021

Zhang, X., Lv, L., Min, G., Wang, Q., Zhao, Y., & Li, Y. (2021). Overview of the Complex Figure Test and Its Clinical Application in Neuropsychiatric Disorders, Including Copying and Recall. *Frontiers in Neurology*, *12*. https://doi.org/10.3389/fneur.2021.680474
